# The pleiotropic regulation of cyclin D1 by newly identified sesaminol-binding protein ANT2

**DOI:** 10.1038/oncsis.2017.10

**Published:** 2017-04-03

**Authors:** M Watanabe, Y Iizumi, M Sukeno, M Iizuka-Ohashi, Y Sowa, T Sakai

**Affiliations:** 1 Department of Molecular-Targeting Cancer Prevention, Kyoto Prefectural University of Medicine, Kyoto, Japan; 2 Division of Endocrine and Breast Surgery, Kyoto Prefectural University of Medicine, Kyoto, Japan

## Abstract

The expression of cyclin D1 is upregulated in various cancer cells by diverse mechanisms, such as increases in mRNA levels, the promotion of the translation by mammalian target of rapamycin complex 1 (mTORC1) signaling and the protein stabilization. We here show that sesaminol, a sesame lignan, reduces the expression of cyclin D1 with decreasing mRNA expression levels, inhibiting mTORC1 signaling and promoting proteasomal degradation. We subsequently generated sesaminol-immobilized FG beads to newly identify sesaminol-binding proteins. As a consequence, we found that adenine nucleotide translocase 2 (ANT2), the inner mitochondrial membrane protein, directly bound to sesaminol. Consistent with the effects of sesaminol, the depletion of ANT2 caused a reduction in cyclin D1 with decreases in its mRNA levels, mTORC1 inhibition and the proteasomal degradation of its protein, suggesting that sesaminol negatively regulates the function of ANT2. Furthermore, we screened other ANT2-binding compounds and found that the proliferator-activated receptor-γ agonist troglitazone also reduced cyclin D1 expression in a multifaceted manner, analogous to that of the sesaminol treatment and ANT2 depletion. Therefore, the chemical biology approach using magnetic FG beads employed in the present study revealed that sesaminol bound to ANT2, which may pleiotropically upregulate cyclin D1 expression at the mRNA level and protein level with mTORC1 activation and protein stabilization. These results suggest the potential of ANT2 as a target against cyclin D1-overexpressing cancers.

## Introduction

Cyclin D1 is one of the most important cell cycle regulators in the G1 to S phases and is overexpressed in various cancers, such as breast cancer,^1, 2^ non-small-cell lung cancer,^1, 3^ melanoma,^4^ pancreatic cancer^5^ and colorectal cancer.^6^ The causative mechanisms of cyclin D1 overexpression are intricate and diverse at each of the transcriptional, translational and posttranslational levels. For example, various kinds of transcriptional factors of cyclin D1, such as activator protein-1 (AP-1),^7, 8^ Sp1,^9^ nuclear factor-κB,^9^ CREB,^10^ signal transducer and activator of transcription factor 3 (STAT3),^11, 12^ STAT5^13, 14^ and T-cell factor (TCF)/lymphoid enhancer factor^15, 16^ are constitutively activated in cancer cells. Regarding the translational control of cyclin D1, mammalian target of rapamycin complex 1 (mTORC1) signaling is known to promote the translation of cyclin D1 mRNA.^17, 18, 19^ The protein stability of cyclin D1 is then mainly regulated via the ubiquitin proteasome system.^20, 21, 22^ Thus the expression of cyclin D1 is pleiotropically regulated in the progression of cancer.

The screening and exploitation of small-molecule agents, which reduce the expression of cyclin D1, is expected for anticancer treatments and chemoprevention.^23, 24, 25^ Various natural compounds also have been reported to downregulate cyclin D1 in cancer cells.^22, 26, 27, 28^ We herein found that sesaminol, a sesame lignan from sesame oil, accumulated cells in G1 phase and pleiotropically reduced the expression of cyclin D1 at the mRNA and protein levels with mTORC1 inhibition and protein degradation. Furthermore, in an attempt to elucidate the mechanisms by which sesaminol reduces the expression of cyclin D1 in a multifaceted manner, we newly identified the inner mitochondrial membrane protein, adenine nucleotide translocase 2 (ANT2), as a sesaminol direct binding protein using sesaminol-immobilized FG beads.

ANT2 maintains the homeostasis of intracellular ATP levels in the inner mitochondrial membrane.^29, 30, 31^ Meanwhile, ANT2 is overexpressed in proliferative cells,^32^ including cancer cells,^33, 34, 35^ and the oncogenic function of ANT2 has been recently focused and vigorously investigated. For instance, the suppression of ANT2 results in the induction of apoptosis,^34, 35, 36^ indicating that ANT2 functions as an oncoprotein. However, limited information is available on the mechanisms by which ANT2 regulates the cell cycle and cell growth in cancer cells.

In the present study, our chemical biology approach revealed the noteworthy functions of the newly identified sesaminol-binding protein ANT2, which may pleiotropically upregulate cyclin D1 levels at the mRNA and protein levels with the activation of mTORC1 signaling and protein stabilization. Therefore, ANT2 has potential as a target against cyclin D1-overexpressing cancers.

## Results

### The sensitivity to sesaminol corresponds to a reduction in cyclin D1

**Figure 1:**
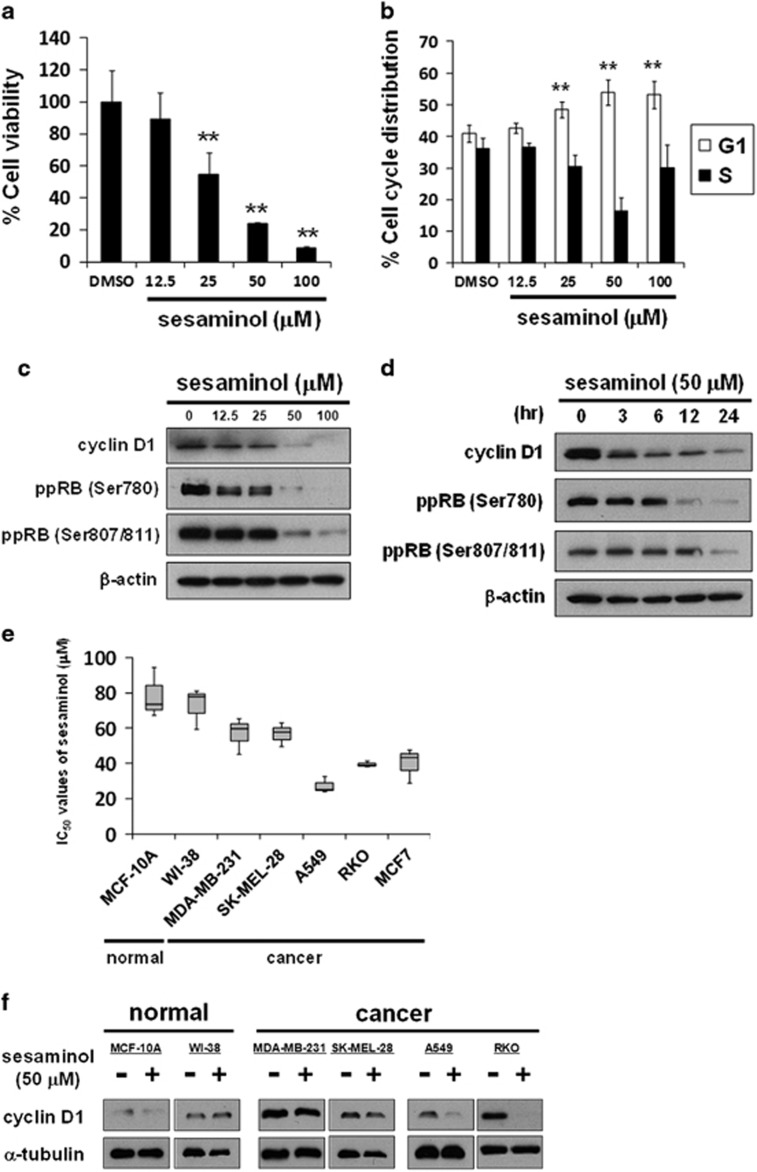
The sensitivity to sesaminol corresponds to a reduction in cyclin D1. (**a**) MCF7 cells were treated with sesaminol at the indicated concentrations for 72 h. Cell viability was measured with a Cell Counting Kit-8 assay. The data obtained with dimethyl sulfoxide (DMSO) are taken as 100%. Columns, means (*n*=3); bars, s.d. **P<0.01, significantly different from the DMSO-treated control. (**b**) MCF7 cells were treated with sesaminol at the indicated concentrations for 24 h. The DNA contents of the cells were determined by flow cytometry. The percentages of cells in the G1 and S phases of the cell cycle are shown. Columns, means (*n*=3); bars, s.d. **P<0.01, significantly different from the DMSO-treated control. (**c**) Cyclin D1 and phosphorylated RB at serine 780 and serines 807/811 were analyzed by western blotting in MCF7 cells treated with sesaminol at the indicated concentrations for 24 h. β-Actin was used as a loading control. (**d**) Cyclin D1 and phosphorylated RB at serine 780 and serines 807/811 were analyzed by western blotting in MCF7 cells treated with 50 μM sesaminol for the indicated times. β-Actin was used as a loading control. (**e**) The IC_50_ values of normal human cell lines (MCF-10 A cells and WI-38 cells) and a variety of human cancer cell lines (MDA-MB-231 cells, SK-MEL-28 cells, A549 cells, RKO cells and MCF7 cells) treated with sesaminol were calculated. The grouped data of IC_50_ values from three independent experiments are shown as box plots. The median value is shown by a horizontal line in the box plot. (**f**) Cyclin D1 was analyzed by western blotting in normal human cell lines and a variety of human cancer cell lines treated with 50 μM sesaminol for 6 h. α-Tubulin was used as a loading control.

Sesaminol from sesame oil has been reported as an antioxidative compound^37, 38, 39^; however, little is known about its anticancer effect. To investigate the effect of sesaminol on cancer cells, we exposed human breast cancer MCF7 cells in the presence of various concentrations of sesaminol for 72 h. As shown in Figure 1a, sesaminol dose-dependently inhibited cell growth. We then investigated how sesaminol affected the cell cycle using flow cytometry. Sesaminol dose-dependently accumulated cells in G1 phase (Figure 1b). We next performed western blotting to analyze the protein expression of cyclin D1, which is one of the most crucial molecules to promote G1–S boundary. Sesaminol reduced the expression of cyclin D1 with dephosphorylation of the RB protein in a dose-dependent manner (Figure 1c). We also observed that sesaminol at 50 μM time-dependently reduced cyclin D1 expression, and RB was subsequently converted to the hypophosphorylated form (Figure 1d). We then treated various cells, including normal and cancer cells with sesaminol. As shown in Figure 1e, the IC_50_ values of sesaminol were relatively higher in normal cells (human lung fibroblast WI-38 cells and normal breast epithelial MCF-10A cells) than in human cancer cells (breast cancer MDA-MB-231 cells, melanoma SK-MEL-28 cells, lung cancer A549 cells, colorectal cancer RKO cells and MCF7 cells). We then treated these cells with 50 μM sesaminol for 6 h to analyze cyclin D1 expression by western blotting. In MCF-10A cells, there was little apparent expression of cyclin D1 with or without sesaminol treatment (Figure 1f). In WI-38 cells, the reduction in cyclin D1 by sesaminol treatment was not observed (Figure 1f). Also in MDA-MB-231 and SK-MEL-28 cells, which showed relatively higher IC_50_ values than other cancer cells, sesaminol treatment did not reduce cyclin D1 expression (Figure 1f). Contrarily, sesaminol remarkably reduced cyclin D1 expression levels in A549 and RKO cells (Figure 1f), in which sesaminol inhibited cell growth similarly to MCF7 cells (Figure 1e). These results suggest that sensitivity to sesaminol corresponds to a reduction in cyclin D1.

### Sesaminol directly binds to ANT2

**Figure 2:**
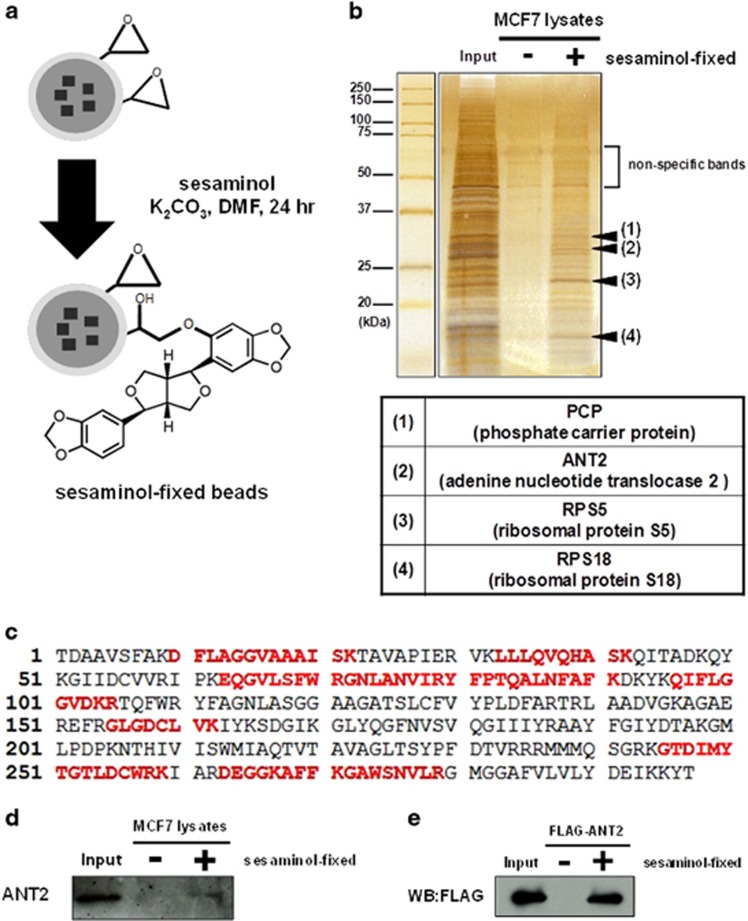
Sesaminol directly binds to ANT2 in MCF7 cells. (**a**) Scheme of fixation of sesaminol onto magnetic FG beads and the estimated structure of sesaminol-fixed beads. (**b**) Four sesaminol-binding proteins were purified from whole-cell extracts of MCF7 cells with sesaminol-fixed (+) or not (−) FG beads and detected by silver staining. Mass spectrometric analysis identified phosphate carrier protein (PCP), ANT2, ribosomal protein S5 (RPS5) and ribosomal protein S18 (RPS18) as sesaminol-binding proteins. (**c**) The amino-acid sequence of ANT2. The residues predicted by mass spectrometry are shown in red. (**d**) Bound ANT2 were detected by western blotting with an anti-ANT2 antibody. (**e**) Purified recombinant FLAG-ANT2 was incubated with sesaminol-fixed (+) or not (−) FG beads and bound FLAG-ANT2 was detected by western blotting with an anti-FLAG antibody.

To further investigate how sesaminol reduced cyclin D1 expression in cancer cells, we undertook the identification of sesaminol-binding proteins by chemical biology approach. We then generated sesaminol-immobilized FG beads, as we previously reported^40, 41^ (Figure 2a). We next incubated sesaminol-immobilized beads with whole-cell extracts of MCF7 cells to purify sesaminol-binding proteins. As a consequence, we newly identified four sesaminol-binding proteins, such as phosphate carrier protein, ANT2, ribosomal protein S5 and ribosomal protein S18 by matrix assisted laser desorption/ionization time-of-flight mass spectrometric (MALDI-TOF MS) analysis (Figure 2b). Among these proteins, we focused on an inner mitochondrial membrane protein ANT2, as ANT2 has been reported to function as an oncoprotein and be overexpressed in several types of malignant tumors.^33, 34^ As shown in Figure 2c, mass spectrometric analysis predicted the residues of the specific amino-acid sequence of ANT2, and we then confirmed the binding of ANT2 to sesaminol by western blotting with an anti-ANT2 antibody (Figure 2d). Furthermore, we incubated sesaminol-immobilized beads with purified recombinant FLAG-tagged ANT2 protein (FLAG-ANT2) to verify whether sesaminol directly binds to ANT2. As shown in Figure 2e, bound FLAG-ANT2 to the sesaminol-immobilized beads was detected by western blotting with an anti-FLAG antibody. These results indicate that sesaminol directly binds to ANT2.

### The depletion of ANT2 results in growth inhibition with a reduction in cyclin D1

**Figure 3:**
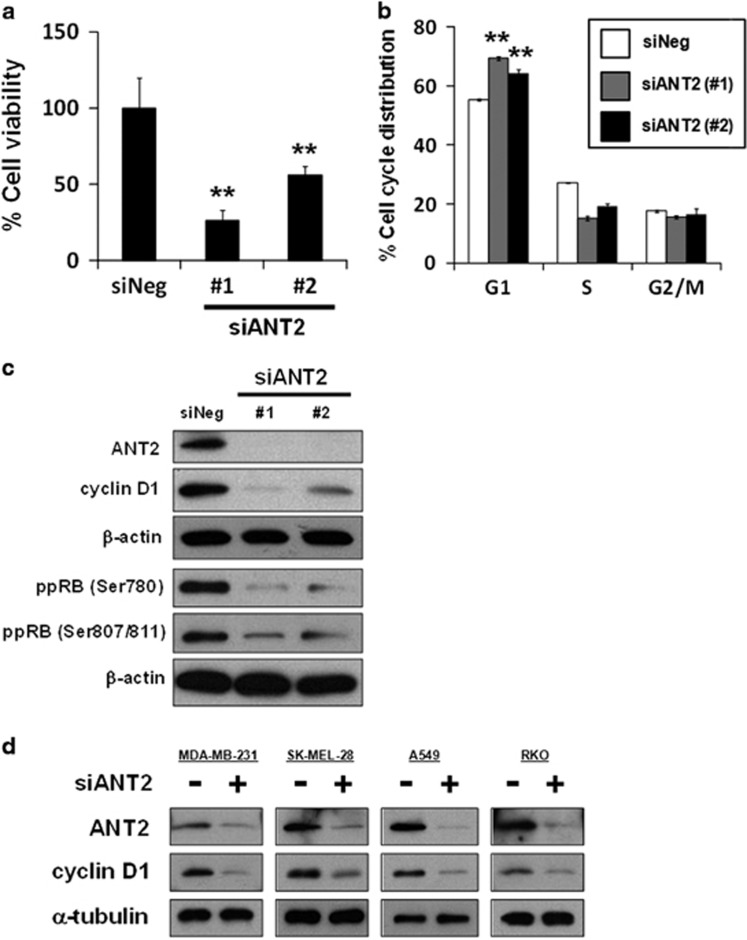
The depletion of ANT2 causes a reduction in cyclin D1 in cancer cells. (**a**) MCF7 cells were treated with two different siRNAs targeting ANT2 (siANT2 #1 and #2) or non-targeting siRNA (siNeg) for 48 h, and the medium was then replaced with the fresh one. After being incubated for 24 h, cell viability was measured with a Cell Counting Kit-8 assay. The data obtained with siNeg are taken as 100%. Columns, means (*n*=3); bars, s.d. **P<0.01, significantly different from the siNeg-treated control. (**b**) MCF7 cells were treated with siANT2 or siNeg for 48 h, and the medium was then replaced with the fresh one. After being incubated for 24 h, the DNA contents of the cells were determined by flow cytometry. The percentages of cells in the G1 and S phases of the cell cycle are shown. Columns, means (*n*=3); bars, s.d. **P<0.01, significantly different from the siNeg-treated control. (**c**) ANT2, cyclin D1 and phosphorylated RB at serine 780 and serines 807/811 were analyzed by western blotting in MCF7 cells treated with siANT2 or siNeg for 48 h. β-Actin was used as a loading control. (**d**) ANT2 and cyclin D1 were analyzed by western blotting in a variety of human cancer cell lines (MDA-MB-231 cells, SK-MEL-28 cells, A549 cells and RKO cells) treated with siANT2 #2 or siNeg for 48 h. α-Tubulin was used as a loading control.

We performed the knockdown of ANT2 in order to determine whether ANT2 regulates cell growth, cell cycle and cyclin D1 expression using two small interfering RNAs (siRNAs) targeting different sequences of the ANT2 gene. The depletion of ANT2 induced growth inhibition (Figure 3a) and accumulated cells in G1 phase (Figure 3b) in MCF7 cells. Consistently, we found a reduction in cyclin D1 with the dephosphorylation of RB after siANT2 treatment (Figure 3c), while the depletion of another major ANT isoform ANT3 did not reduce cyclin D1 expression (Supplementary Figure S1), suggesting that cyclin D1 expression is regulated in an ANT2-specific manner. The reduction in cyclin D1 after siANT2 treatment was also observed in other cancer cell lines, such as MDA-MB-231, SK-MEL-28, A549 and RKO cells (Figure 3d), suggesting that ANT2 functions as an oncoprotein. Taken together, these results suggest that the depletion of ANT2 caused a reduction in cyclin D1 and resulted in the accumulation of G1 cells and growth inhibition, analogous to the treatment with sesaminol binding to ANT2. It should be noted that, although the treatment of sesaminol at 50 μM for 6 h did not reduce cyclin D1 in MDA-MB-231 and SK-MEL-28 cells (Figure 1f), 100 μM sesaminol for 24 or 48 h reduced cyclin D1 in both cell lines (Supplementary Figure S2). Thus these observations are consistent with the hypothesis that sesaminol has the potential to reduce cyclin D1 by binding to ANT2, although the treatment condition of sesaminol appears to be dependent on the cellular context.

### The depletion of ANT2 downregulates cyclin D1 at the mRNA level and protein level with mTORC1 inhibition and protein degradation, similar to the treatment with sesaminol

**Figure 4:**
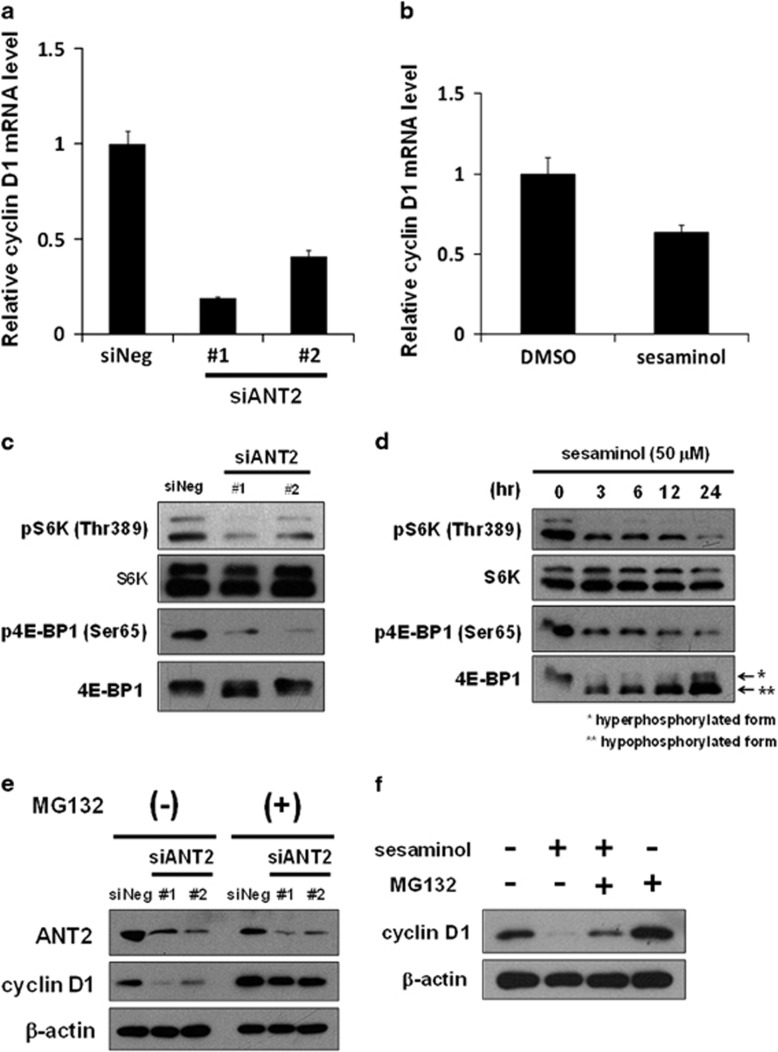
The depletion of ANT2 downregulates cyclin D1 at the mRNA and protein levels with mTORC1 inhibition and protein degradation, analogous to the treatment with sesaminol. (**a**) The expression of cyclin D1 mRNA was measured by real-time RT–PCR in MCF7 cells treated with siANT2 #1, siANT2 #2 or siNeg for 48 h. Cyclin D1 mRNA was normalized to β2MG mRNA, and the data obtained with siNeg are taken as 1.0. Columns, means (*n*=3); bars, s.d. The experiments were carried out three times independently. (**b**) The expression of cyclin D1 mRNA was measured by real-time RT–PCR in MCF7 cells treated with 50 μM sesaminol for 3 h. Cyclin D1 mRNA was normalized to β2MG mRNA, and the data obtained with dimethyl sulfoxide (DMSO) are taken as 1.0. Columns, means (*n*=3); bars, s.d. The experiments were carried out three times independently. (**c**) Phosphorylated S6K at threonine 389, total S6K, phosphorylated 4E-BP1 at serine 65 and total 4E-BP1 were analyzed by western blotting in MCF7 cells treated with siANT2 or siNeg for 48 h. (**d**) Phosphorylated S6K at threonine 389, total S6K, phosphorylated 4E-BP1 at serine 65 and total 4E-BP1 were analyzed by western blotting in MCF7 cells treated with 50 μM sesaminol for the indicated times. (**e**) MCF7 cells were treated with siANT2 or siNeg for 24 h, and the medium was then replaced with that containing 10 μM MG132 or DMSO. After being incubated for 24 h, ANT2 and cyclin D1 were analyzed by western blotting. β-Actin was used as a loading control. (**f**) Cyclin D1 was analyzed by western blotting in MCF7 cells treated with 50 μM sesaminol with or without 10 μM MG132 for 6 h. β-Actin was used as a loading control.

We next investigated the mechanisms by which ANT2 mechanistically regulates cyclin D1 expression. As cyclin D1 expression may be intricately regulated at the transcriptional, translational and posttranslational levels, we initially analyzed the expression level of cyclin D1 mRNA in siANT2-treated cells. The depletion of ANT2 suppressed cyclin D1 mRNA levels (Figure 4a) without a decrease of cyclin D1 promoter activity in the region up to 962 bp from the transcription start site (Supplementary Figure S3). We next investigated whether sesaminol also suppressed the expression of cyclin D1 mRNA. Consistent with the depletion of ANT2, 50 μM sesaminol for 3 h similarly suppressed the expression of cyclin D1 mRNA (Figure 4b).

The translation of cyclin D1 mRNA is known to require mTORC1 signaling.^17, 18, 19^ We examined whether ANT2 also affects mTORC1 signaling. As shown in Figure 4c, the depletion of ANT2 inhibited the phosphorylation of the two major substrates of mTORC1, ribosomal protein S6 kinase 1 (S6K1) and eukaryotic translation initiation factor 4E-binding protein 1 (4EBP1). Similarly, 50 μM sesaminol inhibited the phosphorylation of S6K1 and 4EBP1 in a time-dependent manner (Figure 4d), which coincided with the reduction in cyclin D1 (Figure 1d). These results suggest that both the depletion of ANT2 and sesaminol treatment reduces cyclin D1 levels by inhibiting mTORC1 signaling. Furthermore, we determined whether the two major upstream pathways of mTORC1, the PDK1-AKT and AMPK pathways, were affected by the depletion of ANT2. As shown in Supplementary Figure S4, no alteration was observed in the phosphorylation status of PDK1, AKT or AMPK in siANT2-treated cells.

We next investigated whether ANT2 posttranslationally regulates cyclin D1. As cyclin D1 is degraded by proteasomes, we performed the knockdown of ANT2 with or without the proteasome inhibitor MG132. As shown in Figure 4e, the treatment of MG132 restored the siANT2-mediated reduction in cyclin D1, indicating that ANT2 protects cyclin D1 from proteasomal degradation. Similarly, the treatment of sesaminol with MG132 restored the sesaminol-induced reduction in cyclin D1 (Figure 4f), suggesting that sesaminol facilitates the proteasomal degradation of cyclin D1.

Taken together, these results suggest that ANT2 pleiotropically regulates the expression of cyclin D1 at each level, including mRNA expression, the activation of mTORC1 signaling and protein stabilization.

### Troglitazone indirectly binds to ANT2 and reduces cyclin D1 at the mRNA and protein levels with mTORC1 inhibition and protein degradation

**Figure 5:**
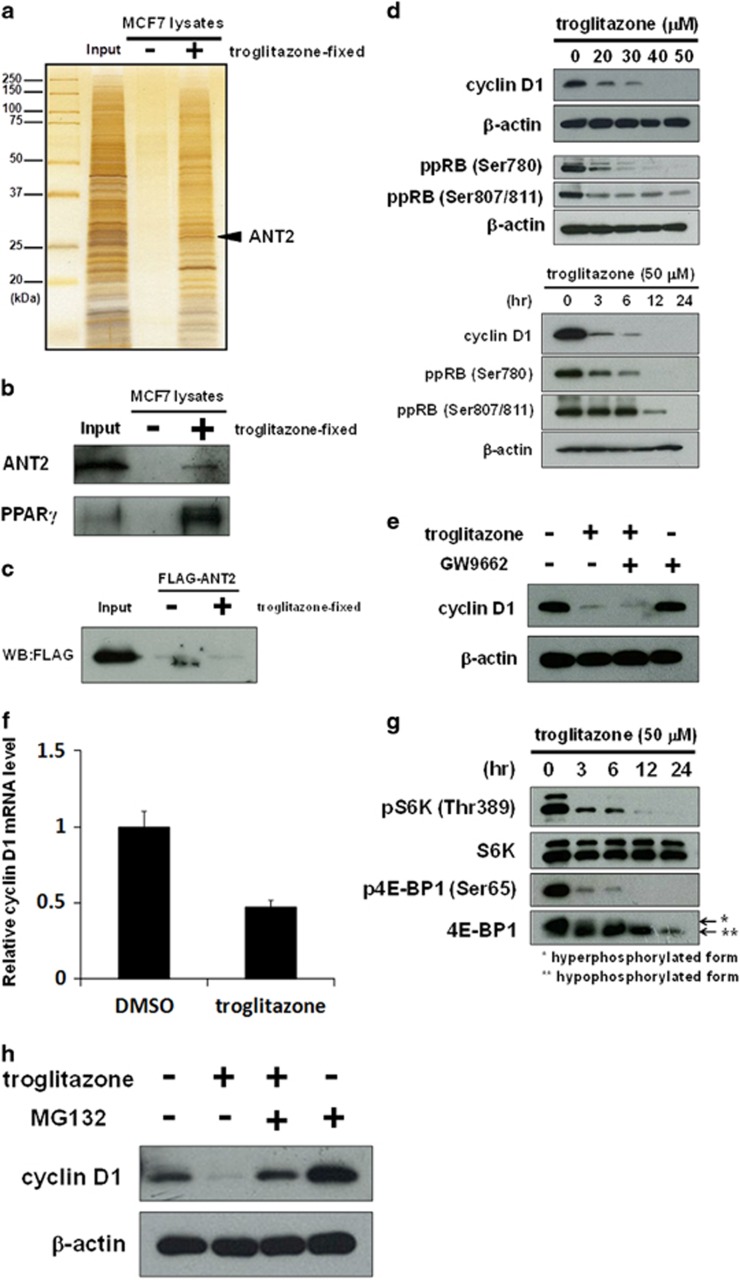
Troglitazone indirectly binds to ANT2 and reduces cyclin D1 at the mRNA and protein levels with mTORC1 inhibition and protein degradation. (**a**) Troglitazone-binding proteins were purified from whole-cell extracts of MCF7 cells with troglitazone-fixed (+) or not (−) FG beads and detected by silver staining. Mass spectrometric analysis identified ANT2 as a troglitazone-binding protein. (**b**) Bound ANT2 and PPARγ were detected by western blotting with anti-ANT2 and anti-PPARγ antibodies. (**c**) Purified recombinant FLAG-ANT2 was incubated with troglitazone-fixed (+) or not (−) FG beads, and bound FLAG-ANT2 was examined by western blotting with an anti-FLAG antibody. (**d**) Upper panel: Cyclin D1 and phosphorylated RB at serine 780 and serines 807/811 were analyzed by western blotting in MCF7 cells treated with troglitazone at the indicated concentrations for 24 h. β-Actin was used as a loading control. Lower panel: Cyclin D1 and phosphorylated RB at serine 780 and serines 807/811 were analyzed by western blotting in MCF7 cells treated with 50 μM troglitazone for the indicated times. β-Actin was used as a loading control. (**e**) Cyclin D1 was analyzed by western blotting in MCF7 cells treated with 50 μM troglitazone with or without 10 μM GW9662 for 24 h. β-Actin was used as a loading control. (**f**) The expression of cyclin D1 mRNA was measured by real-time RT–PCR in cells treated with 50 μM troglitazone for 3 h. Cyclin D1 mRNA was normalized to β2MG mRNA, and the data obtained with dimethyl sulfoxide (DMSO) are taken as 1.0. Columns, means (*n*=3); bars, s.d. The experiments were carried out three times independently. (**g**) Phosphorylated S6K at threonine 389, total S6K, phosphorylated 4E-BP1 at serine 65 and total 4E-BP1 were analyzed by western blotting in MCF7 cells treated with 50 μM troglitazone for the indicated times. (**h**) Cyclin D1 was analyzed by western blotting in MCF7 cells treated with 50 μM troglitazone with or without 10 μM MG132 for 6 h. β-Actin was used as a loading control.

We screened other ANT2-binding compounds in order to investigate whether they also regulate cyclin D1 levels in a manner analogous to the depletion of ANT2. Small-molecule compounds subjected to the screening are as follows: natural compounds (apigenin, quercetin, luteolin, genistein, fucoxanthin, fucoxanthinol, curcumin, epigallocatechin gallate, perillyl alcohol and resveratrol); and medicinal drugs (troglitazone and salicylic acid). We conjugated these compounds to magnetic FG beads, which were then incubated with whole-cell extracts. The purified binding proteins of these compounds were analyzed by MALDI-TOF MS. As a consequence, we found that a dietary flavonoid apigenin and a proliferator-activated receptor-γ (PPARγ) agonist troglitazone were also bound to ANT2. Regarding apigenin, we previously proved that apigenin had the interaction with ANT2 using apigenin-immobilized beads^40^ and expectedly confirmed a dose-dependent reduction in cyclin D1 by apigenin treatment in MCF7 cells (Supplementary Figure S5). Next, as for troglitazone, we confirmed that it also bound to ANT2 (Figure 5a) using troglitazone-immobilized beads (Supplementary Figure S6). We also detected ANT2 and PPARγ by western blotting of the purified troglitazone-binding proteins with anti-ANT2 and anti-PPARγ antibodies, respectively (Figure 5b). However, we could not observe that FLAG-ANT2 bound to troglitazone-immobilized beads (Figure 5c), suggesting that troglitazone bound to ANT2 in an indirect manner. We then investigated whether ANT2 is contributed to the growth inhibitory effects of troglitazone. siANT2-treated MCF7 cells were exposed with 50 μM troglitazone for 72 h. As shown in Supplementary Figure S7, the cell growth was inhibited by troglitazone similarly to siANT2 treatment, and the siANT2 treatment did not further reduce the cell growth in troglitazone-treated cells. These results suggest that ANT2 is a crucial target of troglitazone to inhibit the cell growth. We then confirmed that troglitazone downregulated cyclin D1 levels with the dephosphorylation of RB in dose- and time-dependent manners (Figure 5d), accumulating cells in G1 phase (Supplementary Figure S8) and inhibiting the growth (Supplementary Figure S9). Furthermore, we treated cells with the widely used PPARγ antagonist GW9662 and troglitazone to assess whether the effects of troglitazone are dependent on PPARγ. The concomitant treatment of troglitazone and GW9662 impaired neither the reduction in cyclin D1 (Figure 5e) nor the growth inhibitory effect (Supplementary Figure S9). These results indicate that troglitazone reduces cyclin D1 and inhibits cell growth via a PPARγ-independent mechanism.

We next determined whether the troglitazone-mediated regulation of cyclin D1 is mechanistically similar to the depletion of ANT2. As shown in Figure 5f, 50 μM troglitazone for 3 h reduced the expression of cyclin D1 mRNA, which was similar to the depletion of ANT2 (Figure 4a) and sesaminol treatment (Figure 4b). We also found that 50 μM troglitazone time-dependently inhibited mTORC1 signaling (Figure 5g), which coincided with the reduction in cyclin D1 (Figure 5d, lower panel). Finally, the troglitazone-mediated downregulation of cyclin D1 was restored by the addition of MG132 to the troglitazone treatment (Figure 5h), indicating that troglitazone facilitates the proteasomal degradation of cyclin D1.

Taken together, these results suggest that troglitazone also pleiotropically downregulates cyclin D1 levels by decreasing cyclin D1 mRNA expression levels, inhibiting mTORC1 signaling and promoting proteasomal degradation, analogous to the depletion of ANT2 and sesaminol treatment.

### ANT2 might function in an ATP-independent manner in cancer cells

**Figure 6:**
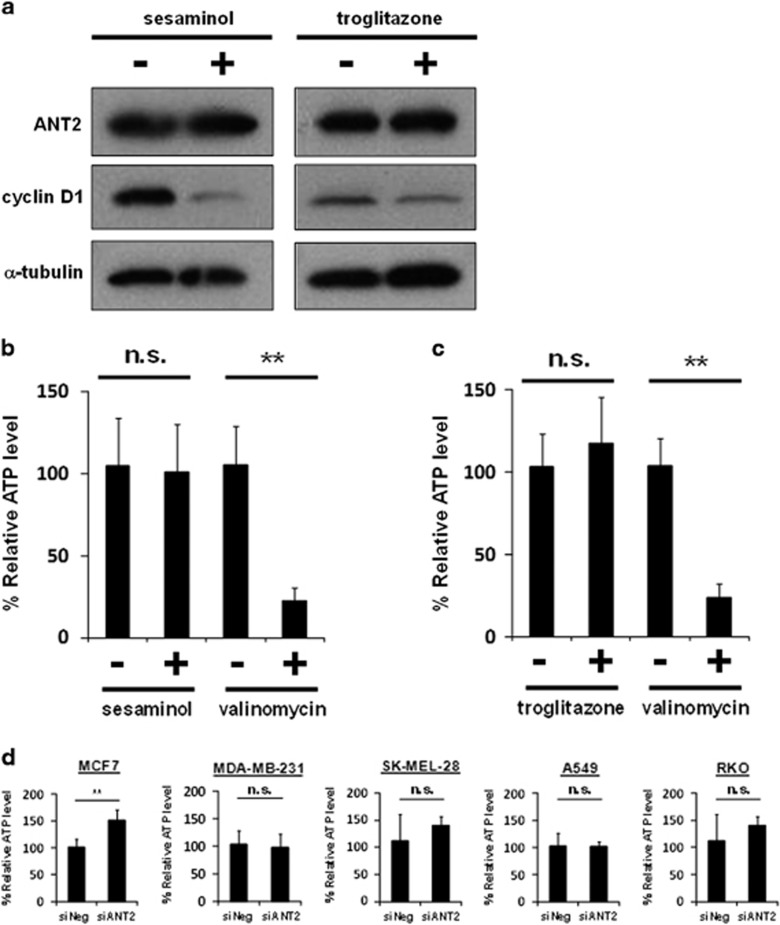
The depletion of ANT2 does not decrease intracellular ATP levels in cancer cells. (**a**) ANT2 and cyclin D1 were analyzed by western blotting in MCF7 cells treated with 50 μM sesaminol or 50 μM troglitazone for 3 h. α-Tubulin was used as a loading control. (**b**) MCF7 cells were treated with 50 μM sesaminol or 5 μM valinomycin for 24 h and then lysed to quantify total intracellular ATP levels. Obtained data were normalized to the absorbance previously measured by a Cell Counting Kit-8 assay. Columns, means (*n*=3); bars, s.d. **P<0.01, significantly different from the dimethyl sulfoxide (DMSO)-treated control. (**c**) MCF7 cells were treated with 50 μM troglitazone or 5 μM valinomycin for 24 h and then lysed to quantify total intracellular ATP levels. Obtained data were normalized to the absorbance previously measured by a Cell Counting Kit-8 assay. Columns, means (*n*=3); bars, s.d. **P<0.01, significantly different from the DMSO-treated control. (**d**) A variety of cancer cells (MCF7 cells, MDA-MB-231 cells, SK-MEL-28 cells, A549 cells and RKO cells) were treated with siANT2 #2 or siNeg for 48 h and then lysed to quantify total intracellular ATP levels. Results were normalized to the absorbance previously measured with a Cell Counting Kit-8 assay. Columns, means (*n*=6); bars, s.d. **P<0.01, significantly different from the siNeg-treated control; NS, not significant.

We investigated how sesaminol and troglitazone inhibited ANT2 function in the downregulation of cyclin D1. First, neither sesaminol nor troglitazone reduced the expression levels of ANT2 in MCF7 cells, although they reduced cyclin D1 expression (Figure 6a). Next, as ANT2 has been implicated in intracellular ATP homeostasis^29, 30, 31^ and is reported to increase intracellular ATP in some types of cancer cells,^33, 34, 35^ we then surmised that sesaminol and troglitazone reduced the intracellular ATP levels by inhibiting ANT2 in cancer cells. Contrary to our expectation, neither of them reduced the intracellular ATP levels in MCF7 cells, while the chemical uncoupler valinomycin, used as a positive control, decreased intracellular ATP levels (Figures 6b and c). Similarly, the depletion of ANT2 did not decrease intracellular ATP levels in various kinds of cancer cells (Figure 6d). These results suggest that the ANT2 regulates cyclin D1 expression independently of intracellular ATP levels in cancer cells.

## Discussion

**Figure 7:**
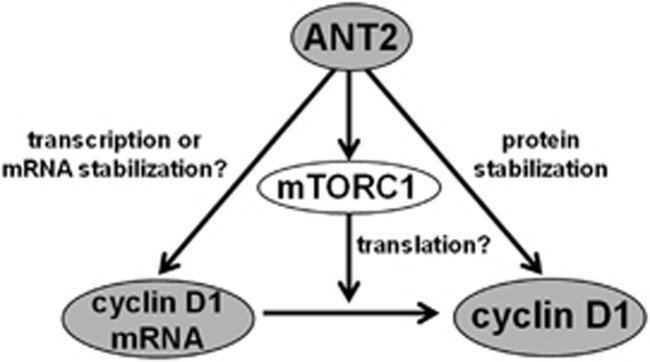
Schematic representation of the pleiotropic regulation of cyclin D1 by ANT2. ANT2 regulates cyclin D1 at the mRNA level (for example, promoting transcription or stabilization of cyclin D1 mRNA) and at the protein level with mTORC1 activation (for example, promoting translation) or protein stabilization.

The overexpression of cyclin D1 is intricately regulated at the mRNA and protein levels, thereby promoting G1–S cell cycle progression and cell growth. The downregulation of cyclin D1 has the potential in the treatment of cancer, and various compounds that suppress cyclin D1 expression have been investigated. In the present study, we undertook a chemical biology approach and obtained the following novel results: (i) sesaminol reduced cyclin D1 and inhibited cell growth in several types of cancer cells; (ii) sesaminol reduced cyclin D1 at the mRNA and protein levels with mTORC1 inhibition and protein degradation, directly binding to the inner mitochondrial membrane protein ANT2; (iii) siANT2 also negatively regulated cyclin D1 expression at the mRNA and protein levels with mTORC1 inhibition and protein degradation; and (iv) the synthetic PPARγ agonist troglitazone indirectly binds to ANT2 and also reduced cyclin D1 in the same manners as those of sesaminol and siANT2 treatments. Thus these results may reveal not only the unique mechanisms of the cyclin D1 downregulators sesaminol and troglitazone but also the pleiotropic regulation of cyclin D1 by ANT2 (Figure 7).

Sesaminol, a phytochemical from sesame oil, has been focused as an antioxidative compound^37, 38, 39^; however, few studies have reported its anticancer activities.^42, 43^ We found that sesaminol could reduce cyclin D1 in a multifaceted manner, such as downregulating the mRNA and protein levels with mTORC1 inhibition and protein degradation. Furthermore, the purification of sesaminol-binding proteins using magnetic FG beads revealed that sesaminol directly bound to ANT2. We supposed that sesaminol negatively regulated ANT2 function, as the depletion of ANT2 showed a reduction in cyclin D1 in the same manners by which sesaminol reduced cyclin D1 expression (Figure 4). Thus our study revealed the multifaceted regulation of cyclin D1 by sesaminol, the underlying mechanisms of which may be explained by the direct interaction with ANT2, as well as the possibility of sesaminol as a mother compound of anticancer agents for cyclin D1-overexpressing cancers.

Troglitazone reduced cyclin D1 expression in a PPARγ-independent manner (Figure 5e) as previously reported,^44, 45^ suggesting that unknown target molecules of troglitazone exist other than PPARγ. In our study, we clearly showed that troglitazone conjugated to FG beads bound to ANT2 in whole-cell lysates of MCF7 cells (Figure 5b); however, the binding between troglitazone and purified recombinant FLAG-ANT2 was not direct (Figure 5c). Although future studies are required to address whether there are other molecules bridging over between troglitazone and ANT2, troglitazone-mediated pleiotropic regulations of cyclin D1 might be explained by the negative regulation of ANT2, considering that the depletion of ANT2 phenocopied the effect of troglitazone.

In proliferative cells including cancer cells, ANT2 is overexpressed as an oncoprotein.^33, 34, 35^ For example, the suppression of ANT2 has been shown to upregulate phosphatase and tensin homolog^34^ and tumor necrosis factor-related apoptosis-inducing ligand receptor,^40, 46^ or downregulate heat-shock protein 90,^34^ epidermal growth factor receptor,^34^ Ras,^47^ HER2,^48^ phophorylated Akt^49^ and ABCG2.^36^ In our study, we first showed that ANT2 could regulate cyclin D1 expression levels in various cancer cells. ANT2 is overexpressed in cancer cells or cancer tissues as breast,^33^ lung^33, 34^ and colorectal cancer,^33^ which are also known to be cyclin D1-overexpressing cancers.^1, 2, 3, 6^ Consistently, we also showed that siANT2-mediated downregulation of cyclin D1 were observed in breast cancer, melanoma, lung cancer and colorectal cancer cells (Figures 3c and d), all of which are known as cyclin D1-overexpressing cancers, suggesting that ANT2 is involved in the overexpression of cyclin D1.

The detailed mechanisms how ANT2 could regulate cyclin D1 expression appear a little complicated. We found three possibilities of ANT2-mediated cyclin D1 regulation. First, based on our results that the depletion of ANT2 led to a reduction in cyclin D1 mRNA without a reduction in the cyclin D1 promoter activity in the region up to 962 bp from the transcription start site containing AP-1, Ets, STAT3, STAT5, Sp1, TCF and CREB-binding sites^16, 50^ (Figure 4a and Supplementary Figure S3), ANT2 may stabilize cyclin D1 mRNA or regulate the cyclin D1 promoter activity through other promoter regions. Second, ANT2 may activate mTORC1 signaling, which is well known to promote the translation of cyclin D1 mRNA, without the alteration of the major upperstreams of mTORC1, such as the PDK1-AKT pathway or AMPK pathway (Supplementary Figure S4). Given that other molecules, such as tuberous sclerosis complex and so on, regulate mTORC1 signaling, further investigation is needed to clarify the more precise mechanism(s) in the regulation of mTORC1 by ANT2. Finally, ANT2 may promote cyclin D1 protein stabilization, based on the result that the siANT2-mediated downregulation of cyclin D1 was restored by the addition of the proteasome inhibitor MG132 (Figure 4e). Thus we showed the diverse functions of ANT2 in the regulation of cyclin D1 expression, although more study is required to reveal the complete mechanisms.

ANT2 in the inner mitochondrial membrane mediates oxidative phosphorylation and ATP homeostasis^29, 30, 31^; however, ANT2 function may be more intricate than we have known. Although in some types of cancer cells the expression of ANT2 has been supposed to increase intracellular ATP levels,^33, 34, 35^ our results showed that the depletion of ANT2 as well as sesaminol and troglitazone treatments did not decrease intracellular ATP levels (Figures 6b–d). Consistent with these data, the depletion of ANT2 did not affect the phosphorylated status of AMPK (Supplementary Figure S4), which is known to be activated by the loss of intracellular ATP.^51, 52, 53^ A recent paper reported that the energy metabolism and intracellular ATP levels were dependent on Sirt4–ANT2 interplay in the mitochondria.^31^ Given that the expression or activity of Sirt4 would vary depending on the cellular context, it may be difficult to assess ANT2 activity only by measuring intracellular ATP levels. Furthermore, other report demonstrated that ANT2 as well as another major ANT isoform ANT3 did not mediate mitochondrial ATP/ADP exchange in cancer cells.^54^ Thus ANT2-mediated ATP/ADP exchange appears to be dependent on cellular context, and cyclin D1 expression may be regulated by a noncanonical ANT2 function independently of intracellular ATP levels in cancer cells, although further investigation should be required to unravel the more precise mechanism.

In summary, we herein found novel functions of ANT2 in the regulation of cyclin D1 using a chemical biology approach to identify sesaminol-binding proteins. Our results not only provide insights into the unique molecular basis of ANT2 but also indicate the potential of screening for ANT2-binding compounds as a platform for drug discovery against cyclin D1-overexpressing cancers.

## Materials and methods

### Reagents

Sesaminol was obtained from Nagara Science (Gifu, Japan). Troglitazone, MG132 and GW9662 were obtained from CALBIOCHEM (San Diego, CA, USA). Apigenin was obtained from Wako Pure Chemical Industries, Ltd. (Osaka, Japan). Valinomycin was obtained from Sigma (Saint Louis, MO, USA). These reagents were dissolved in the solvent dimethyl sulfoxide as stock. Purified recombinant protein of *Homo sapiens* ANT2 (SLC25A5) was obtained from OriGene (TP308949; Rockville, MD, USA).

### Cell culture

Human breast cancer cell lines MCF7 and MDA-MB-231, human melanoma cell line SK-MEL-28 and human lung cancer cell line A549 were obtained as the cell lines of NCI-60 from the NCI Developmental Therapeutics Program. Human colorectal cancer cell line RKO, human lung fibroblast cell line WI-38 and normal breast epithelial cell line MCF-10A were obtained from the American Type Culture Collection (Manassas, VA, USA). The authenticity of each cell line was confirmed by short tandem repeat profiling at each cell bank. All cell lines were confirmed to be negative for mycoplasma infection using MycoAlert Mycoplasma Detection Kit (Lonza, Rockland, ME, USA). All cells except for MDA-MB-231 cells were cultured in Dulbecco’s modified Eagle’s medium supplemented with 10% fetal bovine serum, 4 mM glutamine, 50 U/ml penicillin and 100 μg/ml streptomycin. MDA-MB-231 cells were cultured in RPMI-1640 supplemented with 10% fetal bovine serum, 4 mM glutamine, 50 U/ml penicillin and 100 μg/ml streptomycin. Cells were incubated at 37 °C in a humidified atmosphere of 5% CO_2._

### Cell viability assay

The number of viable cells was measured by a Cell Counting Kit-8 assay (Dojindo, Kumamoto, Japan) according to the manufacturer’s instructions. Briefly, after the incubation of cells treated with each drug or siRNA, kit reagent WST-8 was added to the medium and incubated for 4 h. The absorbance at 450 nm of the samples was measured using a multi-plate reader (Molecular Devices, LLC., Sunnyvale, CA, USA). All experiments shown were replicated at least twice.

### Cell cycle analysis

Cells treated with each drug or siRNA were harvested by trypsinization. After centrifugation, cells were suspended in phosphate-buffered saline containing 0.1% Triton X-100 and 25 μg/ml propidium iodide. Stained cells were analyzed using FACSCalibur (Becton Dickinson, Franklin Lakes, NJ, USA). Data were analyzed using the Modfit LT software (Becton Dickinson). All experiments shown were replicated at least twice.

### Protein isolation and western blotting

Cells were lysed with a lysis buffer containing 50 mM Tris-HCl, 1% sodium dodecyl sulfate (SDS), 1 mM dithiothreitol (DTT) and 0.43 mM 4-(2-Aminoethyl) benzenesulfonyl fluoride hydrochloride (ABSF). The lysates were sonicated and centrifuged at 20 400 *g* at 4 °C for 20 min, and the supernatant was collected. Equal amounts of the protein extract were subjected to sodium dodecyl sulfate-polyacrylamide gel electrophoresis (SDS-PAGE) and transferred to a polyvinylidene difluoride membrane (EMD Millipore, Billerica, MA, USA). The following were used as the primary antibody: mouse anti-human cyclin D1 monoclonal antibody (K0062-3; MBL, Nagoya, Japan), mouse anti-human β-actin monoclonal antibody (A5441; Sigma), mouse anti-human α-tubulin monoclonal antibody (CP06; CALBIOCHEM), mouse anti-human ANT2 polyclonal antibody (H00000292-B01P; Abnova, Taipei City, Taiwan), mouse anti-DDK(FLAG) monoclonal antibody (TA50011-100; Origene), rabbit anti-human ANT3 polyclonal antibody (ab85929; Abcam, Cambridge, UK), rabbit anti-human PPARγ polyclonal antibody (sc-7196; Santa Cruz Biotechnology, Inc., Dallas, TX, USA), rabbit anti-human Phospho-Rb (Ser780) (#9307) and Phospho-Rb (Ser807/811) (#9308), rabbit anti-human S6K (#2708) and Phospho-S6K (Thr389) (#9234), rabbit anti-human 4EBP1 (#9644) and Phospho-4EBP1 (Ser65) (#9451), rabbit anti-human AMPK (#2603) and Phospho-AMPK (Thr172) (#2535), rabbit anti-human PDK1 (#3062) and Phospho-PDK1 (Ser241) (#3061) and rabbit anti-human AKT (#9272) and Phospho-AKT (Thr308) (#9275) polyclonal antibodies (Cell Signaling Technology, Inc., Danvers, MA, USA). Signals were detected with Chemi-Lumi One L (Nacalai Tesque, Kyoto, Japan) or Immobilon western Chemiluminescent HRP Substrate (EMD Millipore). All experiments shown were replicated at least twice.

### Preparation of sesaminol- and troglitazone-fixed beads

Magnetic FG beads with epoxy linkers were purchased from Tamagawa Seiki (Nagano, Japan). The beads were incubated with 100 mM sesaminol or 30 mM troglitazone in dimethylformamide containing potassium carbonate at 60 °C for 24 h. After being washed twice with dimethylformamide, the beads were then washed with Milli-Q water.

### Purification and identification of sesaminol- and troglitazone-binding proteins

MCF7 cells were lysed with binding buffer containing 50 mM Tris-HCl, 150 mM NaCl, 1% NP-40, 1 mM DTT and 0.43 mM ABSF at 4 °C for 30 min and centrifuged. The supernatants were used as whole-cell extracts of MCF7 cells. The extracts were incubated with drug-fixed beads or empty beads at 4 °C for 4 h. The beads were washed three times with binding buffer. The bound proteins were eluted with Laemmli dye and subjected to SDS-PAGE. The proteins were stained by aqueous AgNO_3_, and each strip including the protein was cut off to subject to Sequencing Grade Modified Trypsin (Promega, Madison, WI, USA). The peptide fragments from each strip were analyzed using an Autoflex II mass spectrometer (Bruker Daltonics, Billerica, MA, USA) after in-gel digestion. All experiments shown were replicated at least twice.

### RNAi

Oligonucleotides of siRNA for ANT2 and ANT3 were obtained from Dharmacon (Lafayette, CO, USA). The following siRNAs were used: siANT2 #1 (D-007486-03; siGENOME Human SLC25A5 (292) siRNA), 5′-CUGCAGAUAAGCAAUACAA-3′ siANT2 #2 (D-007486-05; siGENOME Human SLC25A5 (292) siRNA), 5′-GCAAUACAAAGGCAUUAUA-3′ siANT3 (M-007487-01; siGENOME Human SLC25A6 (293) siRNA-SMART pool), and a negative control siRNA (D-001210-05; siGENOME Non-Targeting siRNA#5), 5′-UGGUUUACAUGUCGACUAA-3′. Cells were transfected with 30 nM siANT2 or 10 nM siANT3 using Lipofectamine RNAiMAX Reagent (Invitrogen, Carlsbad, CA, USA) according to the manufacturer’s instructions. All experiments shown were replicated at least twice.

### RNA isolation and real-time quantitative reverse transcriptase–PCR (RT–PCR)

Total RNA was isolated from cells treated with each drug or siRNA using Sepasol-RNAI (Nacalai Tesque) according to the manufacturer’s instructions. Total RNA (2 μg) was reversely transcribed to complementary DNA (cDNA) in a 20 μl reaction volume with MMTV-reverse transcriptase (Promega) and oligo (dT) primers (Toyobo, Osaka, Japan). An equivalent volume of cDNA solution was used for quantitative RT–PCR. cDNA was amplified using an ABI 7300 real-time PCR system (Applied Biosystems, Foster City, CA, USA) with TaqMan Probes to CCND1 (Hs00765553_m1) and β2MG (Hs00984230_m1) (Applied Biosystems). The expression of cyclin D1 mRNA was normalized to that of β2MG mRNA in the same sample. All experiments shown were replicated three times.

### ATP assay

Intracellular ATP levels were measured using the CellTiter-Glo 2.0 Assay (Promega) according to the manufacturer’s instructions. Briefly, 24 h after the treatment of each drug, or 48 h after the transfection of siANT2 or negative control siRNA in a 96-well opaque-walled plate, CellTiter-Glo Reagent was added to the medium, and cells were lysed on an orbital shaker for 2 min. After an incubation at room temperature for 10 min, luminescent signal intensity was read on a luminometer (Berthold Technologies USA, LLC, Oak Ridge, TN, USA). Obtained data were normalized to the absorbance measured by a Cell Counting Kit-8 assay, which was performed prior to cell lysis. All experiments shown were replicated at least twice.

### Plasmid DNA transfection and luciferase assay

The full-length promoter plasmid of cyclin D1 (−962CD1) and an empty plasmid (pGL3 Basic) were gifts from O Tetsu, UCSF Helen Diller Family Comprehensive Cancer Center at San Francisco. In all, 0.1 μg each plasmid and 30 nM siANT2 were co-transfected into MCF7 cells using Lipofectamine 2000 (Invitrogen). After 48 h, cells were lysed using MelioraStar-LT (TOYO INK, Tokyo, Japan), and Luciferase activities of the cell lysates were read on a luminometer (Berthold Technologies). Obtained data were normalized to the absorbance measured by a Cell Counting Kit-8 assay, which was performed prior to cell lysis. All experiments shown were replicated at least twice.

### Statistical analysis

All data are presented as the mean±s.d. The significance of differences in the means between three or more groups was tested using a one-way analysis of variance and that of comparisons between two groups was tested using two-tailed unpaired Student’s *t*-test. A value of *P*<0.05 was considered to be significantly different from each control.
