# Supplementary figures and images for: The pleiotropic regulation of cyclin D1 by newly identified sesaminol-binding protein ANT2
Source: Oncogenesis. 2017 Apr 3;6(4):e311–. doi: 10.1038/oncsis.2017.10 (PMC5520487; doi:10.1038/oncsis.2017.10)

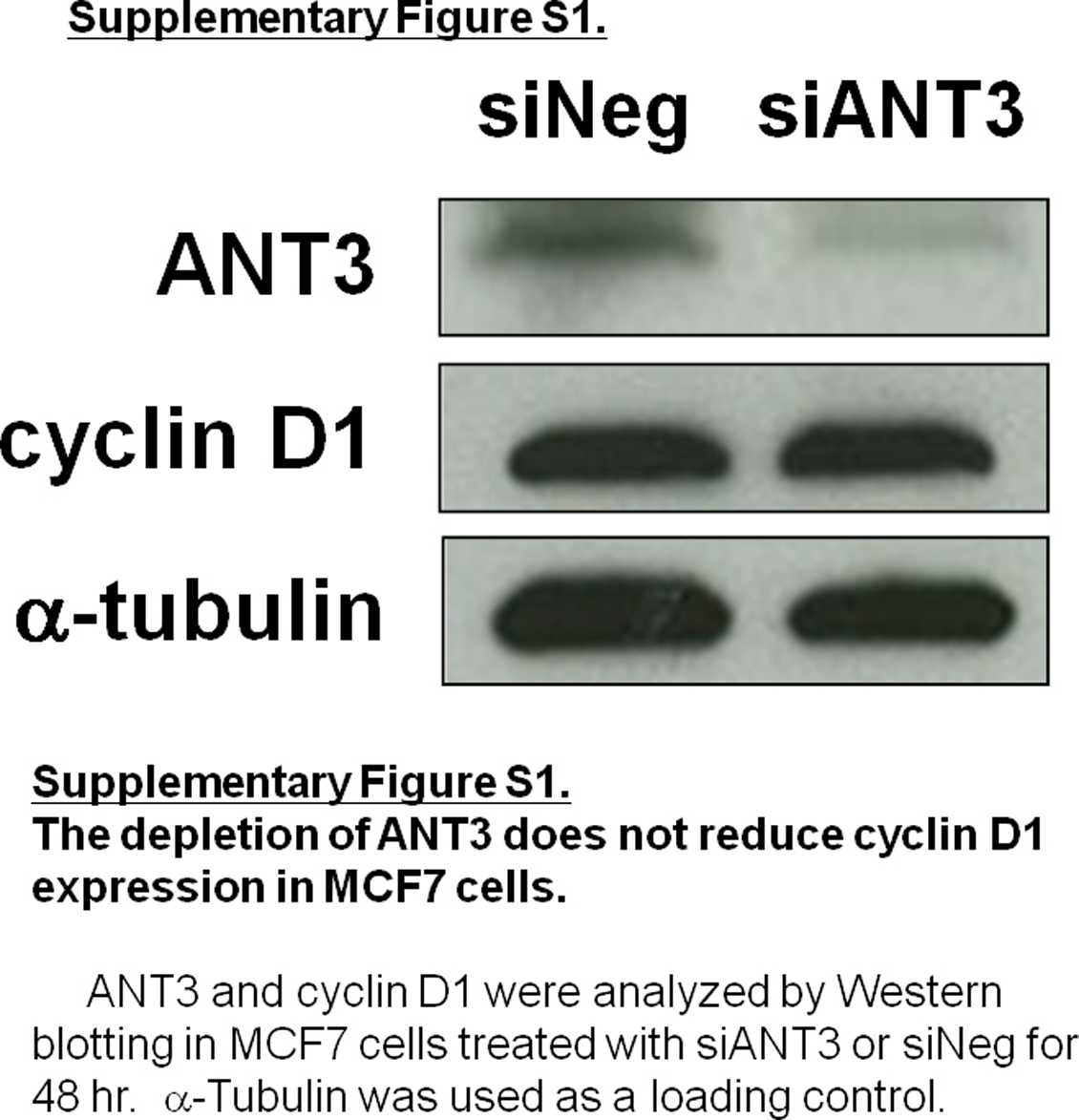

Supplement: Supplementary Figure S1 [file oncsis201710x1.tif]

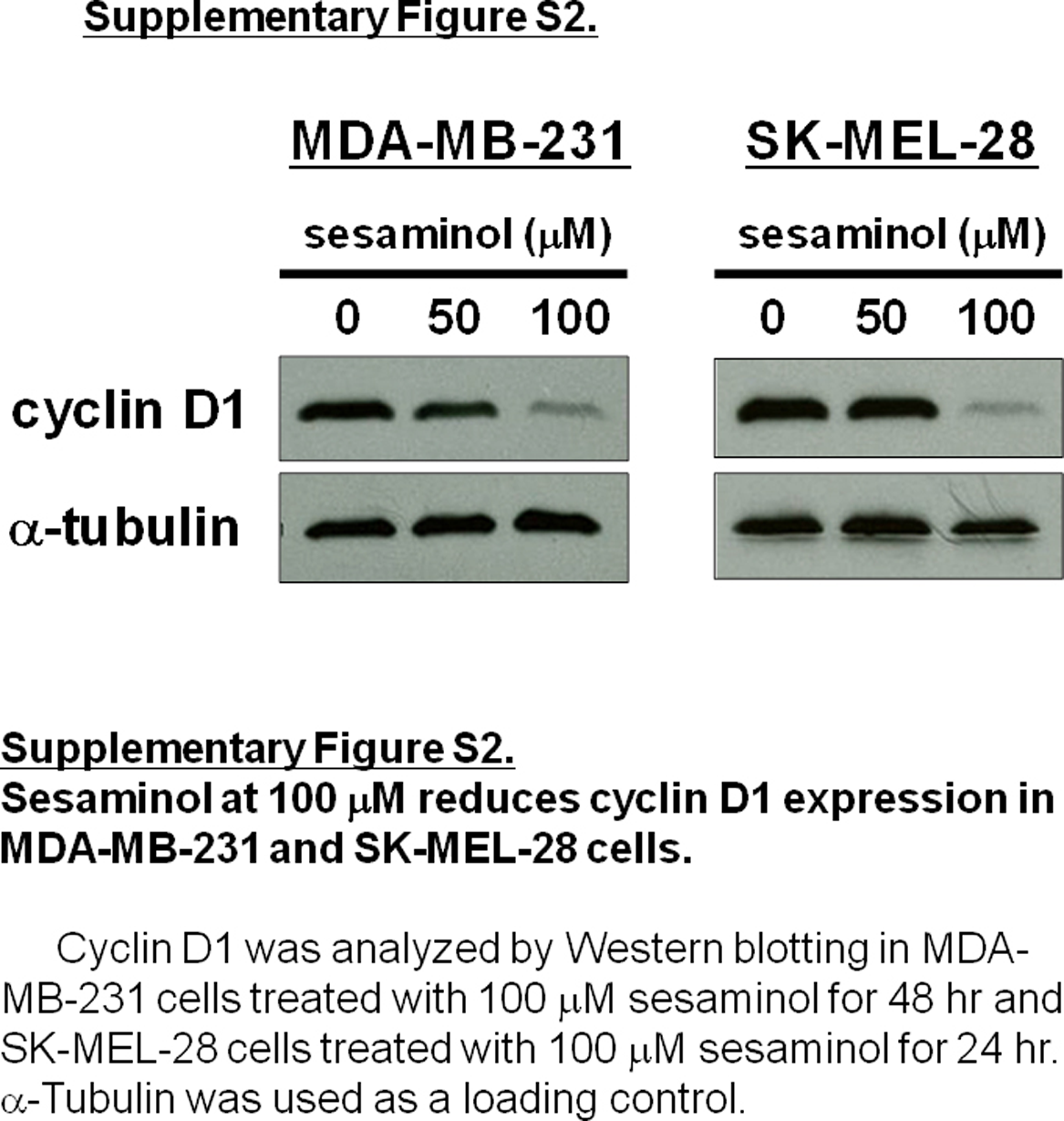

Supplement: Supplementary Figure S2 [file oncsis201710x2.tif]

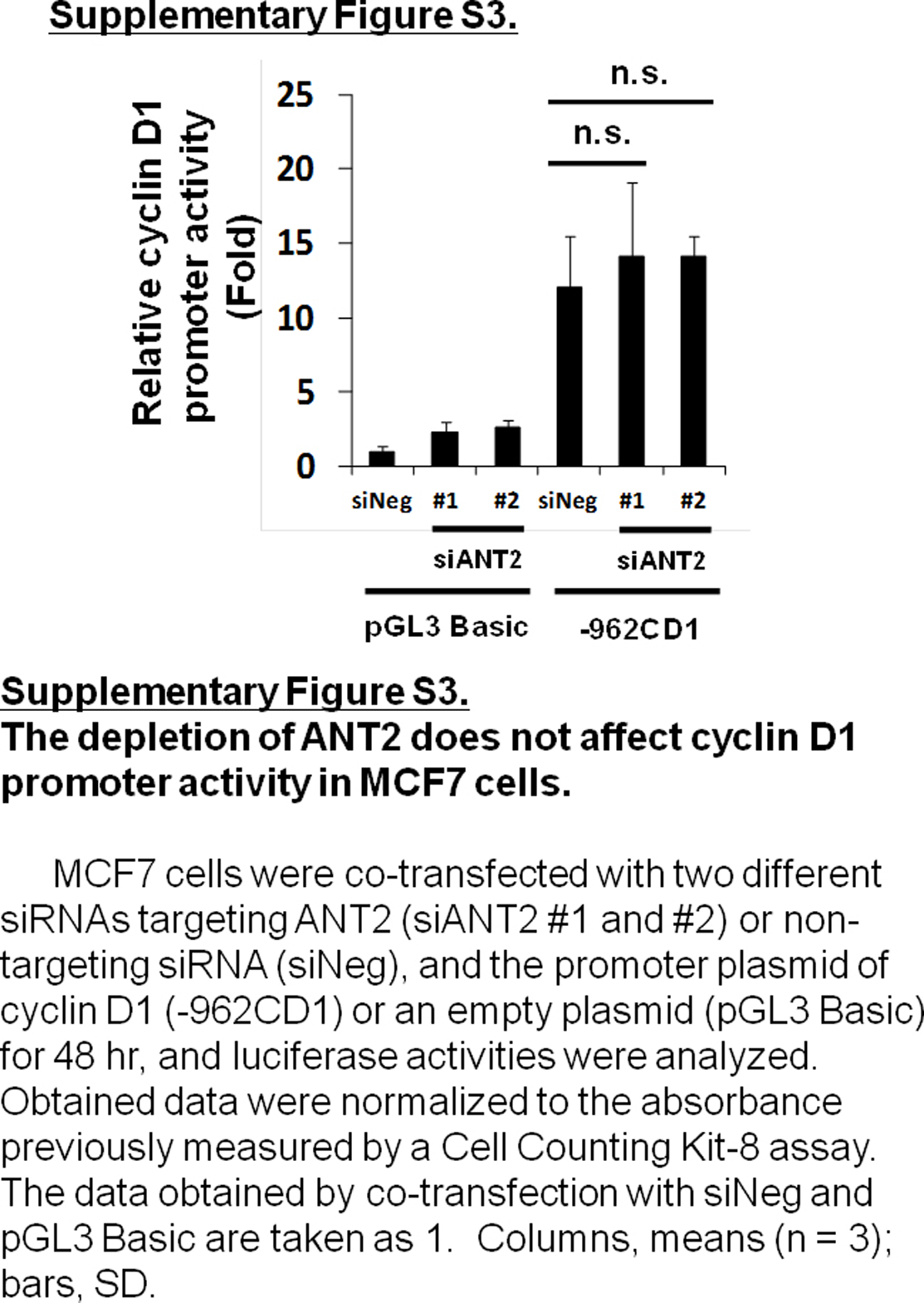

Supplement: Supplementary Figure S3 [file oncsis201710x3.tif]

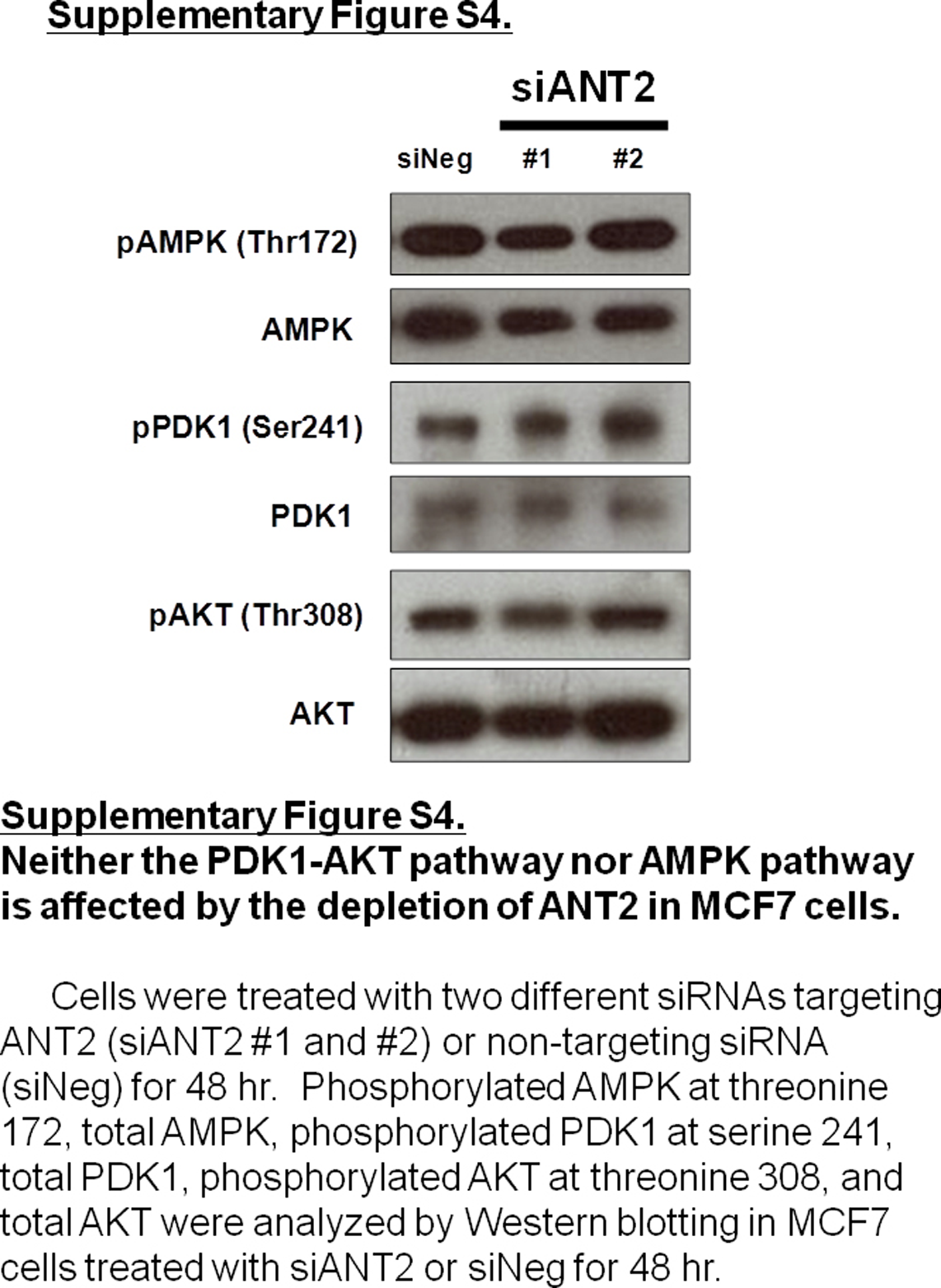

Supplement: Supplementary Figure S4 [file oncsis201710x4.tif]

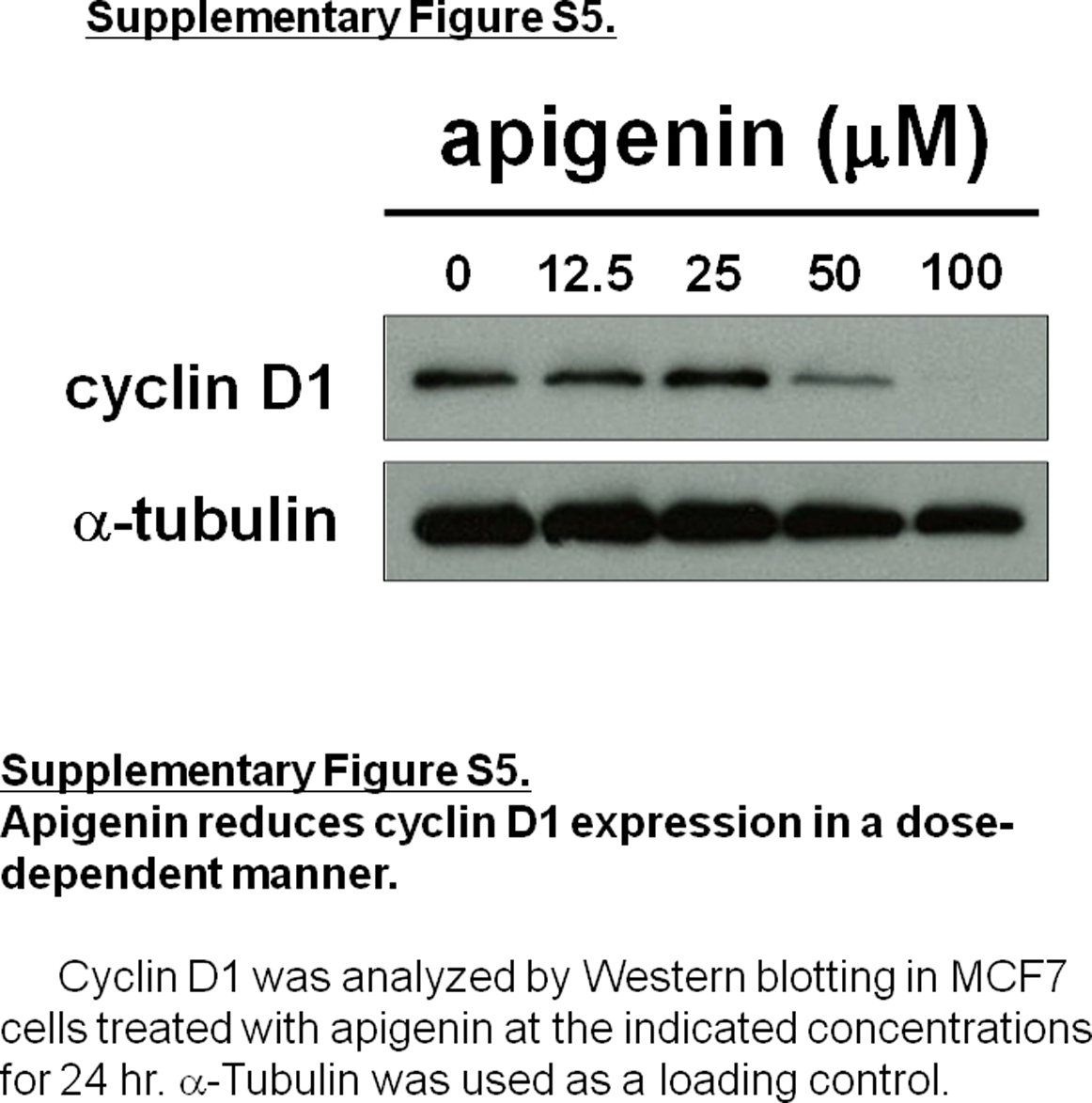

Supplement: Supplementary Figure S5 [file oncsis201710x5.tif]

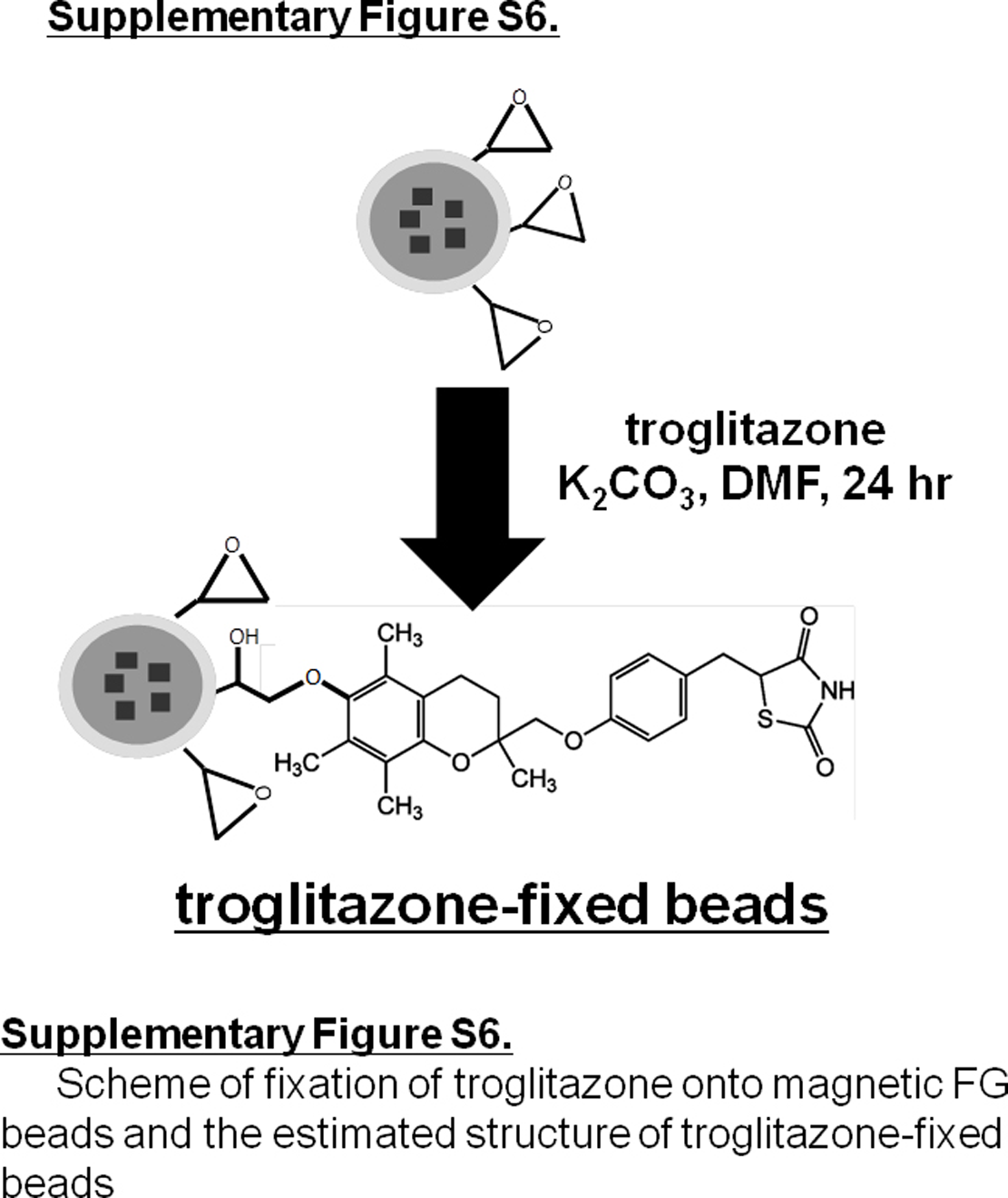

Supplement: Supplementary Figure S6 [file oncsis201710x6.tif]

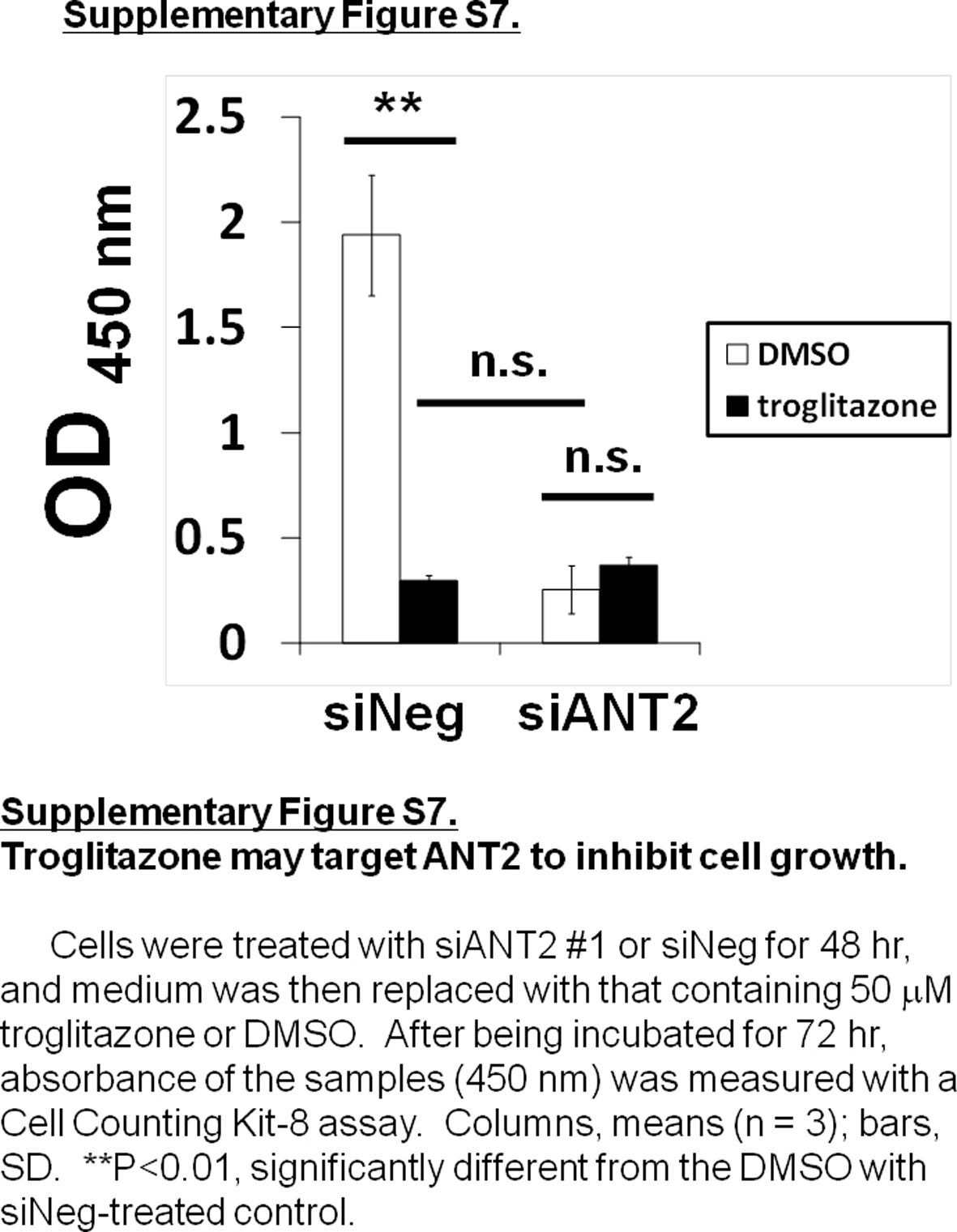

Supplement: Supplementary Figure S7 [file oncsis201710x7.tif]

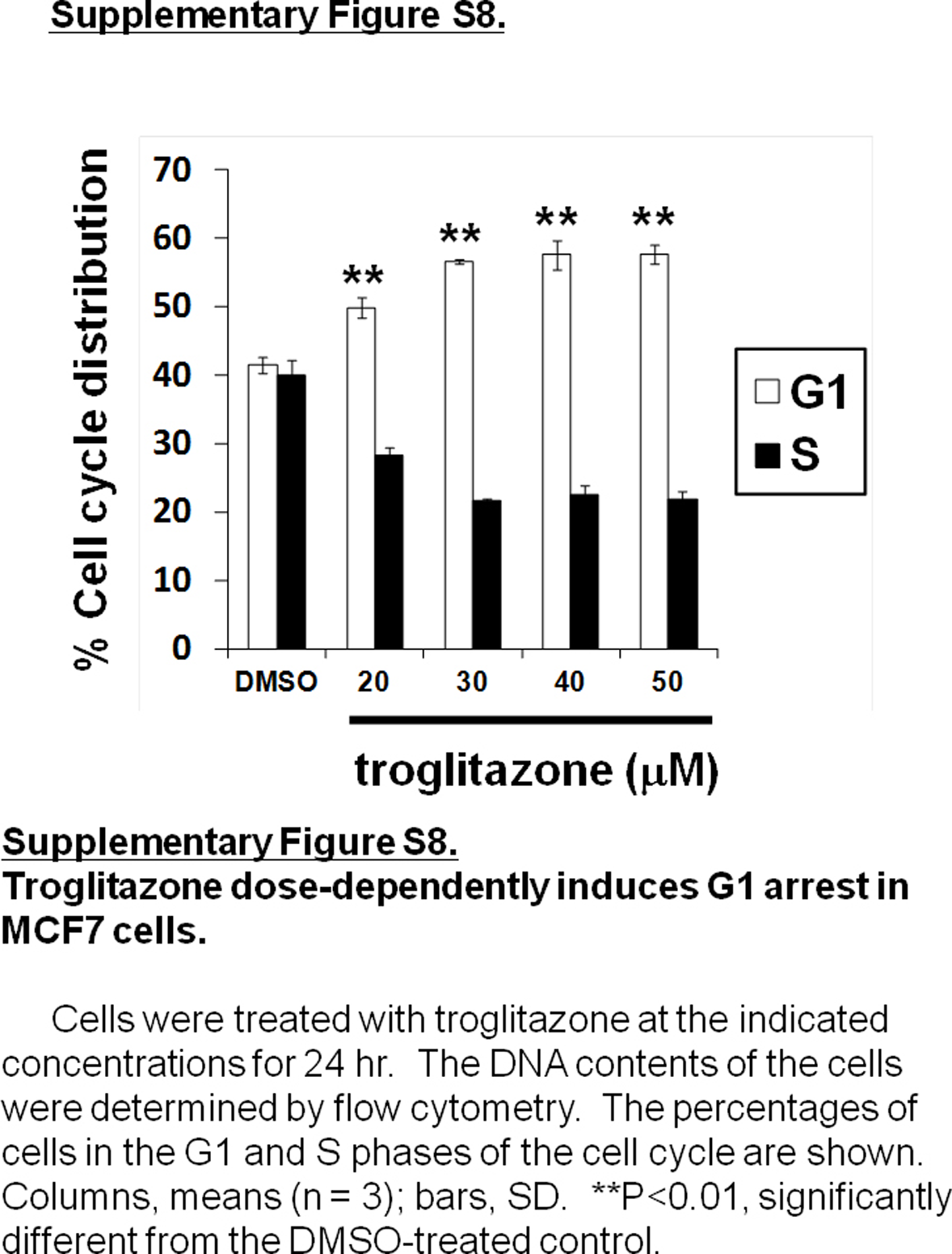

Supplement: Supplementary Figure S8 [file oncsis201710x8.tif]

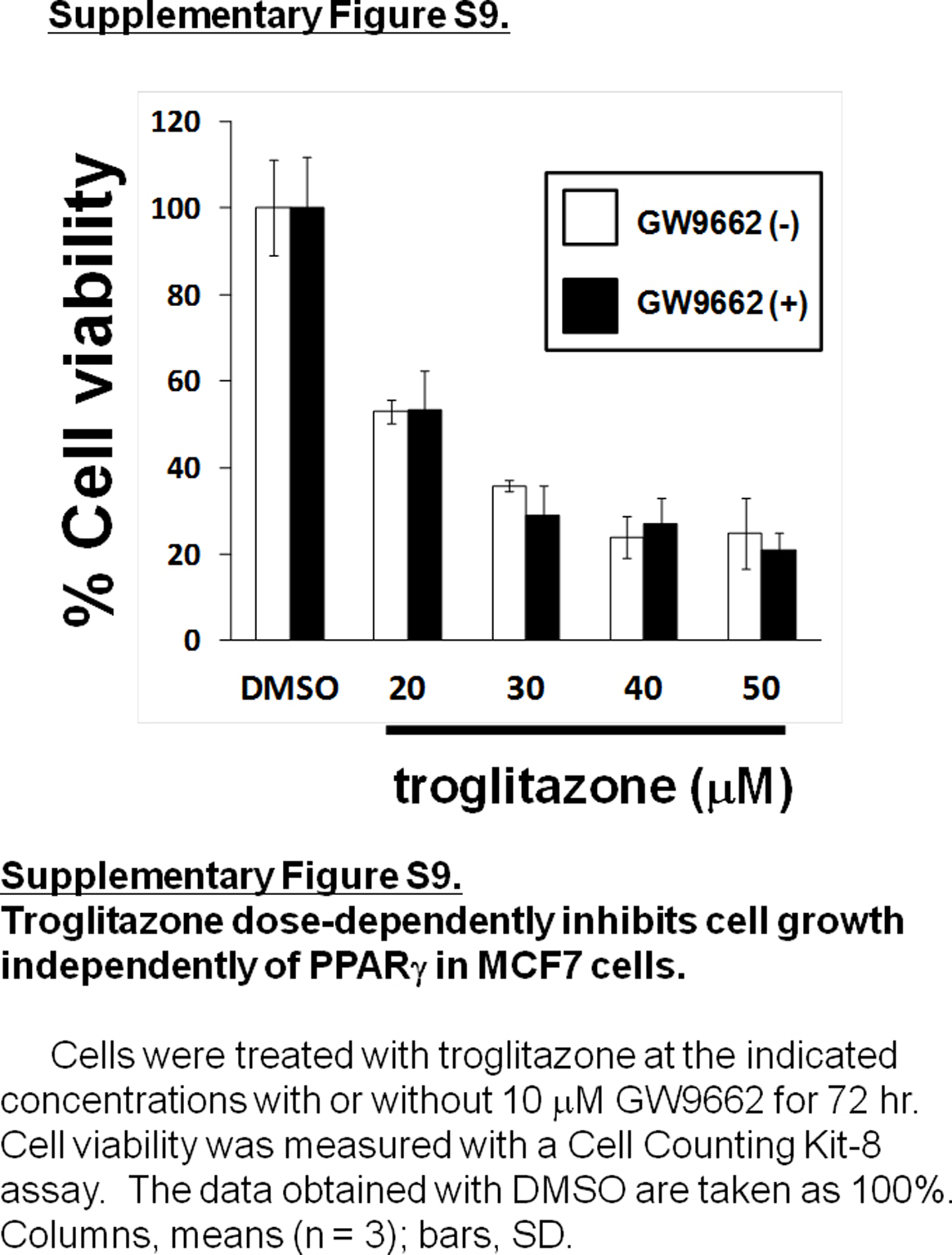

Supplement: Supplementary Figure S9 [file oncsis201710x9.tif]
